# Revealing trends in academic publishing on immersive journalism through a dataset analysis of metaverse and extended technologies from 2017 to 2022

**DOI:** 10.1016/j.dib.2024.110263

**Published:** 2024-03-02

**Authors:** Alberto Sanchez-Acedo, Alejandro Carbonell-Alcocer, Manuel Gertrudix, Jose Luis Rubio-Tamayo

**Affiliations:** Department of Audiovisual Communication and Advertising, Rey Juan Carlos University, Camino del Molino, 5, 28942 Fuenlabrada, Madrid, Spain

**Keywords:** Data analysis, Systematic literature review (SLR), DESLOCIS framework, PRISMA statement, Immersive technologies, Metaverse

## Abstract

This article presents the data obtained from a Systematic Literature Review (SLR) on the use of metaverse and extended technologies for immersive journalism [1]. Boolean operators, both in English and Spanish, were used to retrieve scientific literature using Publish or Perish 8 software on Scopus, Web of Science and Google Scholar between 2017 and 2022. After finding all the scientific literature, a methodological process was carried out using selection criteria and following the PRISMA model to obtain a total sample of 61 scientific articles. The DESLOCIS framework was used for the evaluation and quantitative and qualitative analysis of the retrieved data.

The first dataset [2] contains the metadata of the retrieved publications according to the phases of the PRISMA statement. The second dataset [3] contains the characteristics of these publications according to the DESLOCIS framework.

The data offer the possibility to develop new longitudinal studies and meta-analyzes in the field of immersive journalism.

Specifications Table

**Table:** 

Subject	Social Sciences; Media Technology
Specific subject area	Immersive journalism; Immersive technologies and Metaverse
Data format	Raw Analyzed Filtered
Type of data	.xls files (dataset within tables)
Data collection	The data were obtained after searching in the Publish or Perish 8 [4] software and in the Scopus, Web of Science and Google Scholar databases from 2017 to 2022 using Boolean operators with keywords specific to the research area of study under the eCOMCIENCIA project (PID2021–127019OB-I00). A final sample of data was obtained through PRISMA statement [5] and subsequently analysed using the DESLOCIS framework (Descriptors for a systematic literature review on social sciences) [6] using the LimeSurvey application.
Data source location	Institution: Rey Juan Carlos University City: Madrid Country: Spain Databases and search engine: Web of Science, Scopus and Google Scholar Years: from 2017 to 2022
Data accessibility	[2] Systematic Literature Review Results: PRISMA Statement Phases for Metaverse and Extended Realities in Immersive Journalism. Repository name: Zenodo Direct URL to data: https://doi.org/10.5281/zenodo.7973864 [3] Sample Records: A Systematic Review in Metaverse and Extended Realities in Immersive Journalism. Repository name: Zenodo Direct URL to data: https://doi.org/10.5281/zenodo.7973968
Related research article	[1] A. Sanchez-Acedo, A. Carbonell-Alcocer, M. Gertrudix, J.L. Rubio-Tamayo, Metaverse and Extended Realities in Immersive Journalism: A Systematic Literature Review. Multimodal Technol. Interact. 7 (10), 96 (2023). https://doi.org/10.3390/mti7100096

## 1 Value of the Data

• These data provide a better understanding and identification of the current state of the art of extended technologies and the metaverse applied to journalism. It contains a detailed list of publications that includes the most important milestones in this area.
• The data include the impact of scientific publications in the field under study on the Web of Science, Scopus and Google Scholar, as well as their corresponding metadata. This facilitates the replicability and reproducibility of both this study and research in the field.
• The data reveal a framework in which some gaps and trends in research on the application of immersive technologies to the field of journalism may be found.
• Researchers and journalists may use these data sets in the development of future research. The publication of these datasets encourages the emergence of some complex studies using advanced and metaverse technologies for journalism, such as replication studies, longitudinal studies and meta-analyses.
• The DESLOCIS model provides a framework for the analysis of scientific literature variables in the social sciences applicable to new systematic literature reviews and research in this field.

## 2 Background

The aim of this data article is to present the current state of the art in the field of technologies and the metaverse applied to journalism.

It also aims to facilitate the reproducibility of new studies such as new longitudinal studies, meta-analyzes and systematic literature reviews and, thanks to the literature review, to provide an overview of the state of the art in the field of immersive technologies and the metaverse when applied to immersive journalism. This data article adds value to the corresponding research article by closely examining the data needed for a systematic literature review on this topic.

## 3 Data Description

**Fig. 1:**
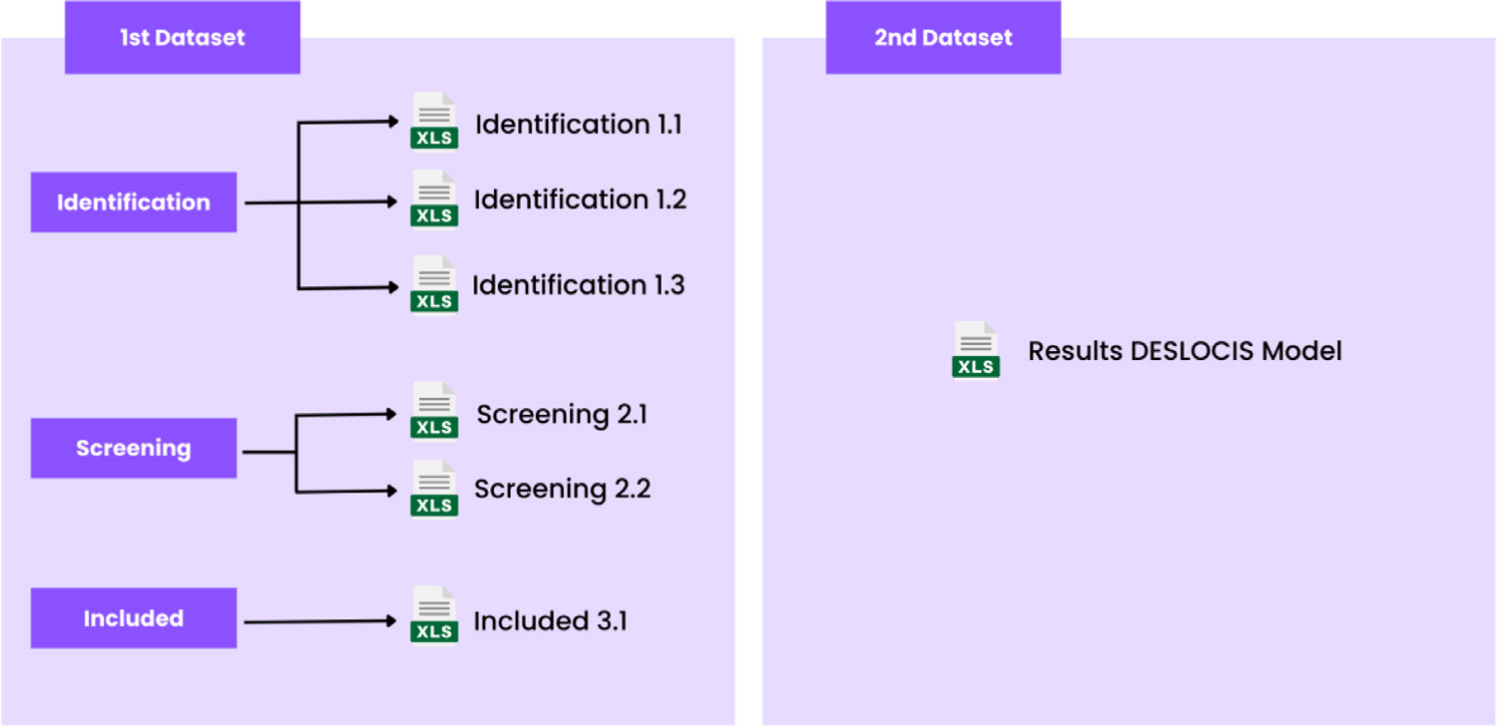
Archives of the datasets presented in this article.

This data article presents two datasets. Fig. 1 shows the two datasets with their respective .xls file. The first dataset contains the .xls file with the results obtained after following the phases proposed in the PRISMA flowchart. The second dataset contains the single .xls file corresponding to the results of the DESLOCIS model.

### 3.1 First dataset

The first dataset contains the metadata of the retrieved publications in .xls files, sorted by rows and columns according to the phases of the PRISMA (Preferred Reporting Items for Systematic Reviews and Meta-Analyzes) statement: Identification, Screening, and Inclusion.

Table 1 describes the overall content of the individual columns that make up the data matrices. The content comes directly from automatic extraction in Web of Science, Scopus and Google Scholar using Publish or Perish 8 software.

**Table 1:** Data identification.

Column	Description
Cites	Number of citations of the article
Authors	Name of the authors of the article
Title	Article title
Year	Year of publication of the article
Source	Journal or publication site
Publisher	Publisher name
ArticleURL	URL
CitesURL	Documents that have cited the paper
GSRank	Result ranking
QueryDate	Document search date
Type	Type of publication (article, conference paper, web, book, book chapter, review…)
DOI	Identifier
ISSN	Journal´s ISSN
CitationURL	URL
Volume Issue	Journal
StartPage	Start page
EndPage	End page
ECC	Estimated Citation Count
CitesPerYear	Cites per year
CitesPerAuthor	Cites per author
AuthorCount	Number of authors
Age	Years of publication
Abstract	Summary of the article
FullTextURL	URL
RelatedURL	URL

#### 3.1.1 Identification phase

For the identification phase of the PRISMA model, 3 .xls files are provided.

The first file is divided into Excel sheets. The first Excel sheet corresponds to the legend to differentiate the results according to the database. The other Excel sheets contain the results of the search using the Boolean operators described in Table 2.

**Table 2:** List of keywords used for Boolean operators in the publication search process.

B1 ENG
“extended reality” AND (“communication” OR “journalism” OR “data representation” OR “audiovisual innovation” OR “audiovisual” OR “innovation” OR “experience” OR “digital content” OR “digital” OR “aframe” OR “a-frame” OR “data visualization” OR “visualization” OR “Immersive journalism” OR “immersion” OR “immersive genres”)
B1 ESP
“realidad extendida” AND (“comunicación” OR “periodismo” OR “representación de datos” OR “innovación audiovisual” OR “audiovisual” OR “innovación” OR “experiencia” OR “contenido digital” OR “digital” OR “aframe” OR “a-frame” OR “visualización de datos” OR “visualización” OR “periodismo inmersivo” OR “inmersión” OR “géneros inmersivos”)
B2 ENG
“XR” AND (“communication” OR “journalism” OR “data representation” OR “audiovisual innovation” OR “audiovisual” OR “innovation” OR “experience” OR “digital content” OR “digital” OR “aframe” OR “a-frame” OR “data visualization” OR “visualization” OR “Immersive journalism” OR “immersion” OR “immersive genres”)
B2 ESP
“XR” AND (“comunicación” OR “periodismo” OR “representación de datos” OR “innovación audiovisual” OR “audiovisual” OR “innovación” OR “experiencia” OR “contenido digital” OR “digital” OR “aframe” OR “a-frame” OR “visualización de datos” OR “visualización” OR “periodismo inmersivo” OR “inmersión” OR “géneros inmersivos”)
B3 ENG
“volumetric video” AND (“communication” OR “journalism” OR “data representation” OR “audiovisual innovation” OR “audiovisual” OR “innovation” OR “experience” OR “digital content” OR “digital” OR “aframe” OR “a-frame” OR “data visualization” OR “visualization” OR “Immersive journalism” OR “immersion” OR “immersive genres”)
B3 ESP
“vídeo volumétrico” AND (“comunicación” OR “periodismo” OR “representación de datos” OR “innovación audiovisual” OR “audiovisual” OR “innovación” OR “experiencia” OR “contenido digital” OR “digital” OR “aframe” OR “a-frame” OR “visualización de datos” OR “visualización” OR “periodismo inmersivo” OR “inmersión” OR “géneros inmersivos”)
B4 ENG
“metaverse” AND (“communication” OR “journalism” OR “data representation” OR “audiovisual innovation” OR “audiovisual” OR “innovation” OR “experience” OR “digital content” OR “digital” OR “aframe” OR “a-frame” OR “data visualization” OR “visualization” OR “Immersive journalism” OR “immersion” OR “immersive genres”)
B4 ESP
“metaverso” AND (“comunicación” OR “periodismo” OR “representación de datos” OR “innovación audiovisual” OR “audiovisual” OR “innovación” OR “experiencia” OR “contenido digital” OR “digital” OR “aframe” OR “a-frame” OR “visualización de datos” OR “visualización” OR “periodismo inmersivo” OR “inmersión” OR “géneros inmersivos”)
B5 ENG
“360° video” AND (“communication” OR “journalism” OR “data representation” OR “audiovisual innovation” OR “audiovisual” OR “innovation” OR “experience” OR “digital content” OR “digital” OR “aframe” OR “a-frame” OR “data visualization” OR “visualization” OR “Immersive journalism” OR “immersion” OR “immersive genres”)
B5 ESP
“360° vídeo” AND (“comunicación” OR “periodismo” OR “representación de datos” OR “innovación audiovisual” OR “audiovisual” OR “innovación” OR “experiencia” OR “contenido digital” OR “digital” OR “aframe” OR “a-frame” OR “visualización de datos” OR “visualización” OR “periodismo inmersivo” OR “inmersión” OR “géneros inmersivos”)
B6 ENG
“360-degree video” AND (“communication” OR “journalism” OR “data representation” OR “audiovisual innovation” OR “audiovisual” OR “innovation” OR “experience” OR “digital content” OR “digital” OR “aframe” OR “a-frame” OR “data visualization” OR “visualization” OR “Immersive journalism” OR “immersion” OR “immersive genres”)
B6 ESP
“video 360” AND (“comunicación” OR “periodismo” OR “representación de datos” OR “innovación audiovisual” OR “audiovisual” OR “innovación” OR “experiencia” OR “contenido digital” OR “digital” OR “aframe” OR “a-frame” OR “visualización de datos” OR “visualización” OR “periodismo inmersivo” OR “inmersión” OR “géneros inmersivos”)
B7 ENG
“360 video” AND (“communication” OR “journalism” OR “data representation” OR “audiovisual innovation” OR “audiovisual” OR “innovation” OR “experience” OR “digital content” OR “digital” OR “aframe” OR “a-frame” OR “data visualization” OR “visualization” OR “Immersive journalism” OR “immersion” OR “immersive genres”)
B7 ESP
“360 video” AND (“comunicación” OR “periodismo” OR “representación de datos” OR “innovación audiovisual” OR “audiovisual” OR “innovación” OR “experiencia” OR “contenido digital” OR “digital” OR “aframe” OR “a-frame” OR “visualización de datos” OR “visualización” OR “periodismo inmersivo” OR “inmersión” OR “géneros inmersivos”)

The second .xls file contains a single matrix with the results of the scientific literature retrieved (*n* = 1291).

The third .xls file is divided into two Excel sheets. The first contains the reviewed, merged results (*n* = 1287) and the second Excel spreadsheet contains the excluded records (*n* = 947).

#### 3.1.2 Screening phase

Two .xls files are provided for the screening phase.

The first consists of two Excel sheets in which the searched data records (*n* = 340) and the excluded data records (*n* = 41) are collected.

The second .xls file contains a single matrix with the reports that were screened for eligibility (*n* = 297).

#### 3.1.3 Included phase

A single .xls file shows the final selection of publications (*n* = 61) after exclusion of invalid records by applying the appropriate exclusion criteria. The second data set after the quantitative and qualitative analysis comes from this file.

### 3.2 Second dataset

The second dataset is an .xls file with the results of the analysis, following the DESLOCIS model, of the selected publications (*n* = 61).

The first Excel sheet shows the results of the DESLOCIS model analysis divided into five sections and the identifiers that allow the presentation to be associated with the record.

The second sheet includes the results of the quantitative and qualitative analysis using the DESLOCIS model.

## 4 Experimental Design, Materials and Methods

To acquire these data, we followed the procedure set out in the Transparent Reporting of Systematic Reviews and Meta-Analyses [7], using the PRISMA flowchart and the PRISMA checklist as key elements to ensure an accurate and standardised systematic review.

The Publish or Perish 8 software and the Web of Science, Scopus and Google Scholar databases were used for literature retrieval. In this software, a series of Boolean operators were established with the Boolean AND and OR following the following formulas, both in Spanish and English:

• General English word” AND (“English specific word” OR “English specific word” OR “English specific word” OR “English specific word” OR “English specific word” [....])
• General Spanish word” AND (“Spanish specific word” OR “Spanish specific word” OR “Spanish specific word” OR “Spanish specific word” OR “Spanish specific word” [....])

The search was carried out using keywords in Spanish and English. The keywords were selected based on the descriptors of the project (IND2022/SOC-23503) funded by the Community of Madrid and on the professional criteria of the researchers, experts in the area under study under the eCOMCIENCIA project (PID2021-127019OB-I00). Table 2 shows the formulas for the search of publications.

The search, which began in October 2022, was limited to literature from the last five years (2017–2022). The five-year period was set for two reasons: firstly, because it is an emerging discipline within journalism studies [8], and secondly, because the initial prospective searches that were conducted found that no studies were found within this period that were clearly focused on the object of study. The total number of results is 1789.

**Fig. 2:**
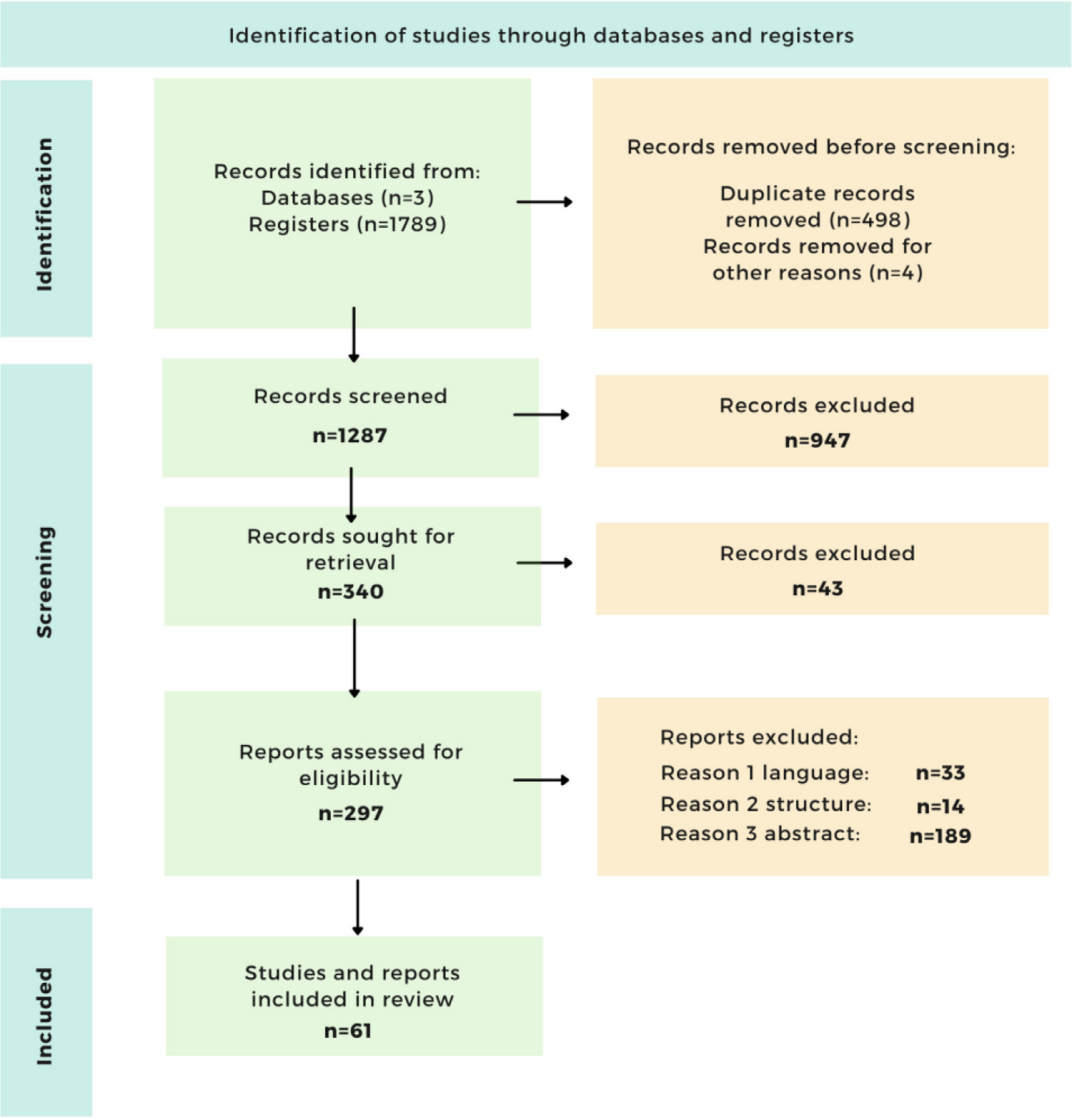
PRISMA statement flow diagram.

Fig. 2 specifies the PRISMA diagram flow that has been followed.

After the 1789 articles were found in the three databases, the duplicate documents (498) and 4 others which, after a second manual check, were also identified as duplicates, were eliminated. These documents include articles, conference papers, book chapters, books, websites and reviews, citations, letters and reviews, surveys, editorials).

For the screening phase, and after eliminating the duplicates and 4 others that were manually eliminated as they were also identified as duplicates, we have a total of 1287 documents, of which we eliminated 947 because we want to keep conference papers and articles. Therefore, we are left with 340 conference papers and articles. These documents are searched one by one and 297 are retrieved, excluding 43 because the link was not found.

Of these 297, 236 were excluded according to the following criteria:

­ 33 by language as we only searched in Spanish or English.
­ 14 were eliminated because they were not articles or conference papers.
­ And 189 have been eliminated because, after reading the abstract, they are not related to the area under study.

Finally, for the studies and reports included in the review, we obtained a total of 61.

These 61 articles have been analysed following the DESLOCIS framework (Descriptors for a systematic literature review on social sciences) using the LimeSurvey application.

For a correct analysis of the results, we have worked with dynamic tables in the Excel application (Table 3).

**Table 3:** shows the number of publications retrieved in each of the searches.

	B1 ENG	B1 ESP	B2 ENG	B2 ESP	B3 ENG	B3 ESP	B4 ENG	B4 ESP	B5 ENG	B5 ESP	B6 ENG	B6 ESP	B7 ENG	B7 ESP
Scopus	59	1	43	11	2	0	49	1	3	1	36	0	36	3
WoS	35	0	24	9	2	0	68	0	1	1	23	0	8	0
GS	89	18	285	25	5	0	334	45	14	0	48	0	276	234
TOTAL	183	19	352	45	9	0	451	46	18	2	107	0	320	237

## Limitations

The results provided in this data article have been obtained by searching specific databases and using specific keywords. When comparing this study with other research related to the subject and with greater characteristics, other databases are considered that are not included in this data article.

With the choice of these keywords and when establishing the search formulas with Boolean operators, results have been obtained that on certain occasions are not pertinent to the area of study or simply have not obtained the desired results.

The criteria established for the searches follow a rigorous methodological process to guarantee the replicability and reproducibility of the study. When carrying out systematic literature review studies it is necessary to point out that there are cases of documentary silence, nevertheless the most important scientific databases and search engines have been used.

## Ethics Statement

The Rey Juan Carlos University gave its approval and authorisation to carry out the study through the approval of the Ethics Committee (Authorisation ID 3105202214722), which ruled in favour of carrying out the research.

Anonymisation of the data is not necessary, as these are scientific publications.

## CRediT authorship contribution statement

**Alberto Sanchez-Acedo:** Conceptualization, Methodology, Investigation, Data curation, Writing – original draft. **Alejandro Carbonell-Alcocer:** Methodology, Investigation, Data curation, Writing – original draft. **Manuel Gertrudix:** Conceptualization, Methodology, Supervision, Writing – review & editing, Project administration, Funding acquisition. **Jose Luis Rubio-Tamayo:** Conceptualization, Supervision, Writing – review & editing, Project administration, Funding acquisition.

## Data Availability

Sample Records: A Systematic Review in Metaverse and Extended Realities in Immersive Journalism (Original data) (Zenodo). Systematic Literature Review Results: PRISMA Statement Phases for Metaverse and Extended Realities in Immersive Journalism (Original data) (Zenodo).
